# Regulation of hepcidin expression by inflammation-induced activin B

**DOI:** 10.1038/srep38702

**Published:** 2016-12-06

**Authors:** Yohei Kanamori, Makoto Sugiyama, Osamu Hashimoto, Masaru Murakami, Tohru Matsui, Masayuki Funaba

**Affiliations:** 1 Division of Applied Biosciences, Graduate School of Agriculture, Kyoto University, Kyoto 606-8502, Japan; 2 Laboratory of Veterinary Anatomy, Kitasato University School of Veterinary Medicine, Towada 034-8628, Japan; 3 Laboratory of Experimental Animal Science, Kitasato University School of Veterinary Medicine, Towada 034-8628, Japan; 4 Laboratory of Molecular Biology, Azabu University School of Veterinary Medicine, Sagamihara 252-5201, Japan

## Abstract

Activin B is induced in response to inflammation in the liver and enhances hepcidin expression, but the source of activin B and the molecular mechanism underlying hepcidin induction are not clear yet. Lipopolysaccharide (LPS)-induced inflammation induced inhibin βB but not inhibin α or inhibin βA expression in the liver, implicating activin B induction. Immunoreactive inhibin βB was detected in endothelial cells and Kupffer cells in LPS-treated liver. Activin B, but not activin A or activin AB, directly increased hepcidin expression. Activin B induced phosphorylation and activation of Smad1/5/8, the BMP-regulated (BR)-Smads. The stimulation of hepcidin transcription by activin B was mediated by ALK2 and ActRIIA, receptors for the TGF-β family. Unexpectedly, activin B-induced hepcidin expression and BR-Smad phosphorylation were resistant to the effects of LDN-193189, an ALK2/3/6 inhibitor. ALK2 and ActRIIA complex formation in response to activin B may prevent the approach of LDN-193189 to ALK2 to inhibit its activity. Activin B also induced phosphorylation of Smad2/3, the TGF-β/activin-regulated (AR)-Smad, and increased expression of connective tissue growth factor, a gene related to liver fibrogenesis, through ALK4 and ActRIIA/B. Activin B-induced activation of the BR-Smad pathway was also detected in non-liver-derived cells. The present study reveals the broad signaling of activin B, which is induced in non-parenchymal cells in response to hepatic inflammation, in hepatocytes.

Hepcidin, a liver-derived peptide hormone, regulates systemic iron homeostasis. Iron overload induces the expression of hepcidin, which binds to and degrades ferroportin, the only known iron exporter, leading to a reduction in serum iron concentrations^1^,^2^. Hepcidin expression is also induced by inflammation, and hepcidin is suggested to be responsible for inflammation-induced anemia^3^. Iron overload and inflammation are thought to be central regulators of hepcidin expression^4^,^5^. Hepcidin expression is primarily regulated at the transcriptional level, whereby BMP6, a member of the BMP subgroup of the TGF-β superfamily that is produced in response to iron overload, increases hepcidin transcription^6^,^7^. Other members of the BMP subgroup, including BMP2, BMP4 and BMP9, also up-regulate hepcidin expression^8^. By contrast, interleukin (IL)-6, which is produced in response to inflammation^9^, plays a role in the inflammation-induced transactivation of hepcidin^5^,^10^.

The TGF-β family is divided into three subgroups: TGF-β, activin and BMP subgroups. All members signal in a similar fashion, whereby the ligands bind to specific type I receptors and type II receptors; type I receptors are called activin receptor-like kinases (ALKs), and type II receptors consist of TβRII, ActRIIA, ActRIIB and BMPRII. Ligand and receptor complex formation activates a type I receptor serine/threonine kinase, leading to the phosphorylation of a receptor-regulated (R)-Smad. Phosphorylated R-Smad forms a complex with Smad4 and regulates target gene transcription^11^,^12^,^13^. The current signaling model indicates that the type I receptor and type II receptor for the TGF-β subgroup are ALK5 and TβRII, respectively, and the activin subgroup signals through the type I receptor ALK4/7 and the type II receptors ActRIIA and ActRIIB. The type I receptors of the BMP subgroup are ALK2/3/6, and the type II receptors are ActRIIA, ActRIIB and BMPRII. R-Smads are categorized as activin/TGF-β pathway-specific R-Smads (AR-Smad: Smad2 and Smad3) and BMP pathway-specific R-Smads (BR-Smad: Smad1, Smad5 and Smad8). Members of the TGF-β and activin subgroups signal via AR-Smads, whereas those of the BMP subgroup signal via BR-Smads^11^,^12^,^13^.

BMP pathway-mediated hepcidin transcription is also involved in inflammation-induced hepcidin transcription. The hepcidin expression induced in the liver in response to lipopolysaccharide (LPS), a potent stimulator of inflammation, was decreased in IL-6 null mice, but still responded to LPS^14^. In addition, treatment with LDN-193189, an inhibitor of ALK2/3/6 that suppresses BR-Smad-mediated signaling^15^, decreased hepatic hepcidin expression in rats and mice with experimentally induced anemia of inflammation^16^,^17^. These results suggest that a molecule(s) eliciting BMP signaling is produced during inflammation to induce hepcidin expression.

Previously, Jones *et al*.^18^ and Besson-Fournier *et al*.^19^ revealed the genetic induction of inhibin βB in LPS-treated liver. In addition, Besson-Fournier *et al*.^19^ showed that activin B, a homodimer of inhibin βB and a member of the activin subgroup of the TGF-β family^20^, stimulated hepcidin transcription through BMP pathway activation. ALK3 transmits the activin B-mediated signal to up-regulate hepcidin expression^19^. These results suggest that activin B is one molecule responsible for inflammation-induced hepcidin expression via the BMP pathway. Consistent with these results, Canali *et al*.^21^ also reported that activin B stimulated the BMP-mediated signaling pathway and hepcidin transcription in Hep3B hepatocytes. However, the mechanism underlying activin B-induced hepcidin expression is controversial. Canali *et al*.^21^ suggested ALK2 as a primary signal receptor to elicit hepcidin transcription. Furthermore, the source of activin B produced during inflammation is unknown. The objective of this study is to identify (1) the localization of activin B in response to LPS, (2) the regulation of hepatic hepcidin expression by activin B, and (3) the activin B-induced signaling pathway.

## Results

### Activin B is induced in the liver in response to LPS treatment

**Figure 1:**
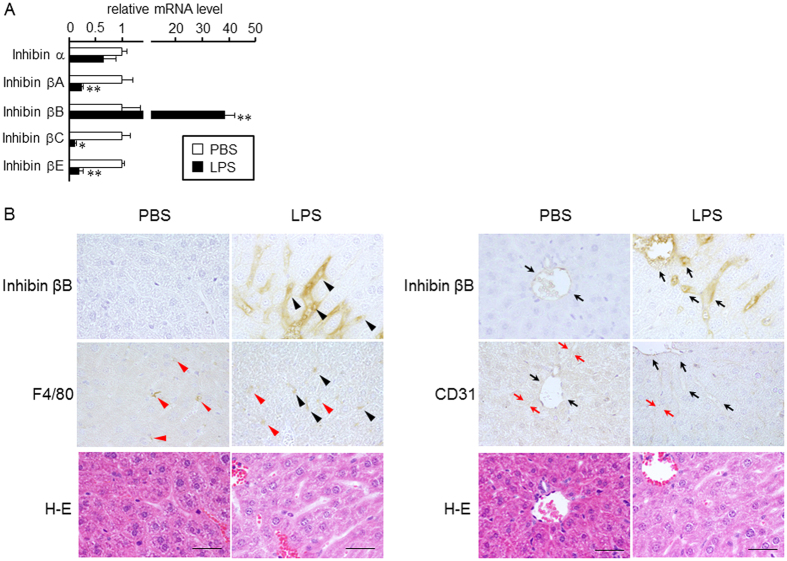
Activin B induction in LPS-treated liver. C57BL/6 mice were intraperitoneally injected with PBS or with LPS (5 mg/kg). At 6 h post-injection, livers were recovered. (**A**) Expression levels of inhibin subunits (**A**) were examined by RT-qPCR analysis, and the expression levels in the control mice were set at 1. Mean ± SE (n = 4). * and **P < 0.05 and *P* < 0.01, respectively, vs. PBS-treated liver. (**B**) Immunolocalization of inhibin βB was examined by immunohistochemistry. A representative result of livers from LPS-treated mice is shown. Upper: localization of inhibin βB-positive cells. Middle: localization of F4/80- or CD31-positive cells. Lower: hematoxylin-eosin (H–E) staining of the serial section of the immunohistochemical image shown above. Arrowheads and arrows indicate Kupffer cells and sinusoidal endothelial cells, respectively. Arrowheads or arrows in black indicate double positive for inhibin βB and F4/80 or inhibin βB and CD31, respectively. Bar: 50 μm.

We first examined the expression and localization of inhibin subunits in LPS-treated livers. Activin is a homo- or heterodimer of the inhibin β subunit, whereas structurally related inhibin is a heterodimer of the inhibin α subunit and β subunits^20^. Inhibin has an opposite effect on follicle-stimulating hormone release from the pituitary to that of activin^22^,^23^, and it does not transmit its signal via Smad^20^. Consistent with previous studies^18^,^19^, LPS induced the hepatic expression of inhibin βB, whereas inhibin βA expression decreased (Fig. 1A). The transcript levels of inhibin βC and βE in the liver were also decreased by LPS, whereas LPS did not affect the expression of inhibin α.

Immunoreactive inhibin βB was detected in some endothelial cells and Kupffer cells resided in central vein, interlobular arteriovenous and sinusoid in LPS-treated liver, whereas faint staining was detected in central vein and interlobular arteriovenous endothelial cells (Fig. 1B, Table 1); inhibin βB-positive cells were also positive for F4/80, a macrophage marker^24^, or CD31, a molecule predominantly expressed in endothelial cells^25^. Furthermore, strong staining was detected in the vessel lumen in LPS-treated livers, and no staining was detected in hepatic stellate cells. Similar results were obtained by centrifugation-based separation of liver cells. Inhibin βB mRNA was predominantly detected in the Kupffer cell/endothelial cell-rich fraction and endothelial cell-rich fraction (Supplementary Fig. S1A). LPS did not change the immunoreactivity of anti-inhibin α or βA antibody significantly. Immunoreactive inhibin α and βA were predominantly detected in hepatocytes and vessel lumen, whereas endothelial cells and Kupffer cells were not stained (Table 1). Taking the regulatory expression and immunolocalization of inhibin subunits in LPS-treated liver together with the gene induction of proinflammatory cytokines (Supplementary Fig. S1B), these results suggest that hepatic inflammation induces the production of activin B, a homodimer of inhibin βB, in vascular endothelial cells and Kupffer cells; the presence of activin B protein should be clarified in future.

### Activin B increases hepcidin expression via BMP-responsive elements but not the STAT-binding site

**Figure 2:**
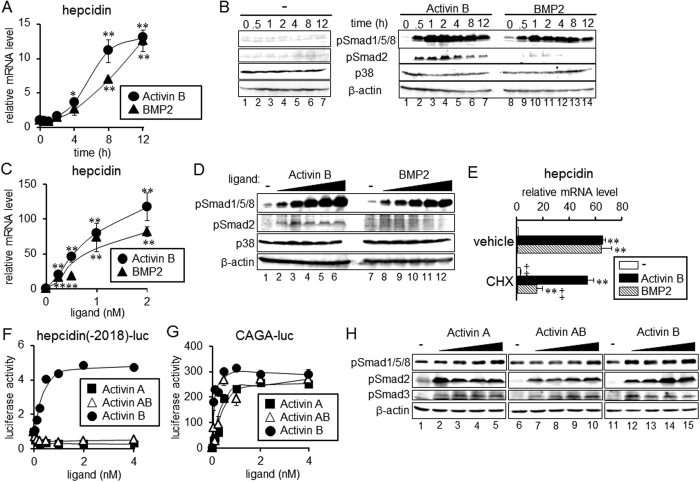
Induction of hepcidin by activin B in rat primary hepatocytes. (**A–D**) Rat primary hepatocytes were cultured in 0.2% FBS for 4 h, followed by treatment with activin B (2 nM) or BMP2 (4 nM) for the indicated time (**A,B**) or by treatment with various concentrations of activin B, i.e., 0, 0.125, 0.25, 0.5, 1 or 2 nM, or BMP2, i.e., 0, 0.25, 0.5, 1, 2 or 4 nM, for 1 h (**D**) or 8 h (**C**). (**A,C**) Hepcidin expression was examined by RT-qPCR analysis. Mean ± SE (n = 3). * and **P < 0.05 and *P* < 0.01, respectively, vs. cells treated without activin B or BMP2. (**B,D**) Phosphorylation of Smad1/5/8 and Smad2 as well as p38 and β-actin as the loading controls was examined by Western blot analysis. (**E**) Effect of cycloheximide was examined. Rat primary hepatocytes were stimulated with activin B (2 nM) or BMP2 (4 nM) in the presence or absence of cycloheximide (1 μg/mL) for 8 h. Hepcidin expression was examined by RT-qPCR analysis. Mean ± SE (n = 3). **P < 0.01 vs. cells treated with the respective inhibitor (vehicle or CHX) but not with activin B or BMP2. ^‡^*P* < 0.01 vs. cells treated with the respective TGF-β family ligand (none, activin B or BMP2) but not with CHX. (**F,G**) HepG2 cells were transfected with the indicated reporters and CMV-βGal. At 4 h post-transfection, cells were treated with or without activin A, activin AB or activin B for 12 h. Luciferase activity normalized to β-galactosidase activity was calculated. Mean ± SE (n = 3). (H) HepG2 cells were treated with increasing concentrations of activin A, activin AB or activin B, i.e., 0, 0.5, 1, 2 or 4 nM, for 1 h. Phosphorylation of Smad1/5/8, Smad2 and Smad3 as well as the loading control β-actin was examined by Western blot analysis. The cropped images of Western blot analysis are shown because of space liminations; images of the full-length blot are Supplementary Fig. S10.

We next examined changes in hepcidin expression with time after treatment with activin B or BMP2 in rat primary hepatocytes. Consistent with previous results^19^,^21^, activin B increased the hepcidin transcript levels (Fig. 2A). Significant up-regulation of hepcidin expression was detected after 4 h of activin B treatment, which was slightly quicker than BMP2-induced hepcidin expression; BMP2 treatment for 4 h did not significantly increase hepcidin expression. Comparable hepcidin induction was detected at 12 h in activin B-treated cells and BMP2-treated cells. BMP-induced hepcidin expression is mediated by phosphorylation and activation of BR-Smad^4^,^5^. Thus, we explored the phosphorylation of Smad1/5/8 by activin B treatment. As shown in Fig. 2B, activin B stimulated the phosphorylation of Smad1/5/8 within 30 min after the treatment, and the phosphorylation continued for at least 12 h; the kinetics of Smad1/5/8 phosphorylation was similar to that seen with BMP2 treatment. In addition to Smad1/5/8 phosphorylation, activin B induced Smad2 and Smad3 phosphorylation. Activin B and BMP2 increased hepcidin expression (Fig. 2C) and Smad1/5/8 phosphorylation in a dose-dependent manner (Fig. 2D); both hepcidin expression and Smad1/5/8 phosphorylation levels increased with increasing concentrations of activin B and BMP2. Treatment with cycloheximide, a protein synthesis inhibitor^26^, did not affect activin B-mediated hepcidin induction, whereas the hepcidin induction by BMP2 was cycloheximide-sensitive (Fig. 2E).

The activin B-induced hepcidin expression was regulated at the transcriptional level. Activin B induced Smad1/5/8 phosphorylation in HepG2 cells with a similar time-course as in primary hepatocytes (Supplementary Fig. S2A), and it stimulated the expression of hepcidin(-2018)-luc in a dose-dependent manner in HepG2 cells (Supplementary Fig. S2B). To evaluate the role of the BMP pathway in activin B-mediated hepcidin transcription, the effects of mutations in the BMP-responsive elements (BMP-REs) in the hepcidin promoter were evaluated. Previous studies have shown that there are two possible BMP-REs: BMP-RE1, spanning nt −155 to nt −150, and BMP-RE2, spanning nt −1678 to nt −1673^27^,^28^. Mutations of either BMP-RE decreased the basal hepcidin transcription, and the fold-induction of activin B stimulation was partially reduced (Supplementary Fig. S2C). Responsiveness to activin B was lost due to mutations of both BMP-RE1 and BMP-RE2, suggesting that similar to BMP, activin B stimulates hepcidin transcription via BMP-REs through the induction of BR-Smad phosphorylation and activation. Hepcidin transcription is also positively regulated via the STAT-binding site (STAT-BS) spanning nt −143 to nt −134^29^, but mutations in the STAT-BS did not change responsiveness to activin B (Supplementary Fig. S2D). Consistent with the results and unlike IL-6, activin B did not stimulate STAT3 phosphorylation (Supplementary Fig. S2E).

### Neither activin A nor activin AB confers hepcidin transcription

Although several dimers of inhibin βB with inhibin subunits are possible, thus far, inhibin B^30^ (a heterodimer of inhibin α and inhibin βB), activin AB^22^ (a heterodimer of inhibin βA and inhibin βB) and activin B^31^ (a homodimer of inhibin βB) have been purified from natural sources. We examined whether similar to activin B, activin A or activin AB induces hepcidin transcription.

Neither activin AB nor activin A stimulated transcription of hepcidin(-2018)-luc within the range of tested concentrations, whereas activin B increased luciferase expression in a dose-dependent manner (Fig. 2F). All three activins stimulated CAGA-luc, a representative activin-responsive reporter mediated by activated AR-Smad^32^ (Fig. 2G). Activin B but not activin A or activin AB induced phosphorylation of Smad1/5/8, whereas all activin isoforms stimulated Smad2/3 phosphorylation with comparable potency (Fig. 2H). These results suggest that homodimerization of inhibin βB is required for the stimulation of hepcidin transcription. Activin A and activin AB were purified from follicular fluid, whereas activin B was purified from cells recombinantly expressing inhibin βB; because it is undeniable that source of activin isoforms affects the results, use of the ligands prepared similarly may be helpful in future studies.

### ALK2 and ActRIIA are responsible for activin B-mediated hepcidin transcription

**Figure 3:**
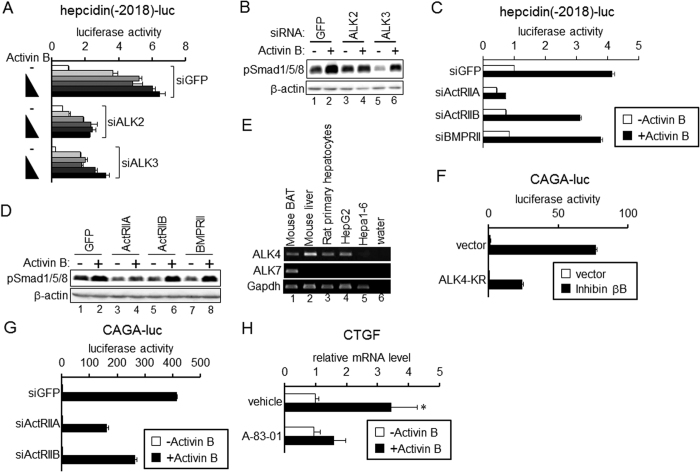
ALK2/ActRIIA and ALK4/ActRIIA/IIB mediate activin B-induced up-regulation of hepcidin and CTGT expression, respectively. (**A–D,G**) HepG2 cells were transfected with siRNA for the indicated gene. At 48 h of transfection, cells were further transfected with the indicated reporter and CMV-βGal (**A**,**C**,**G**). At 4 h of plasmid transfection, cells were treated with or without activin B (A: 0, 0.125, 0.25, 0.5, 1 or 2 nM; C and G: 2 nM) for 12 h. Mean ± SE (n = 3). (**B,D**) At 48 h of post-transfection, cells were treated with or without activin B (2 nM) for 1 h. Phosphorylation of Smad1/5/8 and β-actin was examined by Western blot analysis. (**E**) Expression of receptors for the TGF-β family in the indicated tissues and cells was examined by RT-PCR. (**F**) HepG2 cells transfected with CAGA-luc and CMV-βGal were co-transfected with or without expression vector for inhibin βB (activin B) and the indicated kinase-inactive activin receptor mutant for 16 h. Mean ± SE (n = 3). (**H**) Rat primary hepatocytes were treated with or without A-83-01 (5 μM), followed by treatment with activin B (2 nM) for 8 h. Expression of CTGF was examined by RT-qPCR analysis. Mean ± SE (n = 3). *P < 0.05, vs. cells treated with the respective inhibitor but not with activin B. The cropped images of Western blot analysis and RT-PCR analysis are shown because of space liminations; images of the full-length blot and gel are Supplementary Fig. S11.

Next, we explored which receptors elicit hepcidin transcription induced by activin B. To this end, possible receptors for activin B were individually knocked down by transfection of double-stranded siRNA. Because activin B activated the BMP pathway as shown above and because type I receptors determine signal specificity^11^,^12^,^13^, ALK1, ALK2, ALK3 and ALK6 are suggested to be candidate type I receptors for activin B. Significant expression of ALK1 and ALK6 was not detected in HepG2 cells (data not shown). Decreased expression of ALK2 or ALK3 by transfection with siRNA for ALK2 or ALK3, respectively (Supplementary Fig. S3A), decreased activin B-induced luciferase activity (Fig. 3A). However, ALK3 knockdown decreased the basal transcription of hepcidin, and responsiveness to activin B treatment was not decreased by ALK3 knockdown. Consistent with these results, knockdown of ALK3 decreased basal Smad1/5/8 phosphorylation, but significant increase in Smad1/5/8 phosphorylation in response to activin B was detected (Fig. 3B). By contrast, ALK2 knockdown did not affect the basal transcription of hepcidin (Fig. 3A) or the basal phosphorylation level of BR-Smad (Fig. 3B), but blunted responsiveness to activin B. These results suggest that ALK2 is a type I receptor that confers activin B-induced hepcidin transcription.

We also explored the type II receptors responsible for activin B-induced hepcidin transcription by down-regulating the expression of individual type II receptors (Supplementary Fig. S3B). As shown in Fig. 3C, knockdown of ActRIIA but not ActRIIB or BMPRII blunted the responsiveness of hepcidin transcription to activin B in HepG2 cells. Consistent with these results, activin B-induced phosphorylation of Smad1/5/8 was inhibited by ActRIIA knockdown (Fig. 3D). Hepa1-6 hepatoma cells lack significant expression of ActRIIA (Supplementary Fig. S4A). Although Hepa1-6 cells did not respond to activin B, forced expression of ActRIIA but not TβRII or BMPRII elicited efficient transcription of hepcidin by activin B; ActRIIB expression marginally increased hepcidin transcription (Supplementary Fig. S4B). These results suggest that ActRIIA is a main type II receptor for activin B-induced hepcidin transcription.

### ALK4 and ActRIIs are required for activin B-induced AR-Smad-mediated signaling

Similar to activin A, activin B has been shown to signal via the type I receptor ALK4 and AR-Smad^33^,^34^. In addition, activin B can confer AR-Smad-mediated signaling via ALK7^21^,^33^,^34^. We examined ALK4 and ALK7 expression in liver and liver-derived cells (Fig. 3E). RT-PCR analysis indicated that ALK4 was expressed in the liver, primary hepatocytes and HepG2 cells, whereas the ALK7 expression level was below the detection limit in the liver and liver-derived cells. Activin B-induced CAGA-luc transcription was increased in response to activin B, and the induction was decreased by expression of ALK4(KR), a kinase-inactive mutant of ALK4 that acts as a dominant-negative form^34^ (Fig. 3F). These results suggest that ALK4 is a receptor that confers the signal via AR-Smad. We further explored the type II receptor responsible for activin B-mediated AR-Smad phosphorylation and activation by use of kinase-inactive mutant activin receptor and CAGA-luc. Expression of ActRIIB(KR) decreased the luciferase expression induced by activin B expression, and ActRIIA(KR) had a lesser potency to inhibit activin B-mediated CAGA-luc transcription (Supplementary Fig. S5A). Knockdown of ActRIIA or ActRIIB also decreased responsiveness to activin B to stimulate transcription of CAGA-luc (Fig. 3G). Previous studies have shown that expression of connective tissue growth factor (CTGF), a principle inducer of liver fibrogenesis^35^, is up-regulated by activated AR-Smad in hepatocytes^36^,^37^. To explore the role of activin B-induced phosphorylation of AR-Smad, we evaluated CTGF expression and the effects of A-83-01, an inhibitor of ALK4/5/7, to block AR-Smad-mediated signaling^38^. Activin B increased CTGF expression, and the up-regulation of CTGF expression was inhibited by A-83-01 in primary hepatocytes (Fig. 3H). As expected, A-83-01 blocked activin B-induced phosphorylation of Smad2 and Smad3 in primary hepatocytes (Supplementary Fig. S5B) and the transcription of CAGA-luc in HepG2 cells (Supplementary Fig. S5C).

Provided that activin B elicits BR-mediated signaling via ALK2 and ActRIIA and AR-mediated signaling via ALK4 and ActRIIs, we hypothesized that ALK2 forms complex with ALK4 as well as ActRII. We first evaluated complex formation of endogenous receptors by co-immunoprecipitation assay in HepG2 cells, but could not detect it because of insensitivity of the antibody. Thus, complex formation was examined in HepG2 cells expressed receptor encoding Flag-tagged ALK2, HA-tagged ALK4 or 6Myc-tagged ActRIIA. After cross-linking using BS3, a cell-impermeable cross-linker, cell lysates were immunoprecipitated with anti-Flag antibody, followed by Western blot. ALK2 specifically migrated at ~360 kDa (band 1), ~300 kDa (band 2), and ~140 kDa (band4) in a BS3-dependent manner (Supplementary Fig. S6A). In addition, Western blot analysis indicated the bands at ~360 kDa (band 1), ~300 kDa (band 2) and ~140 kDa (band 4) as the immunoreactive molecules for anti-HA antibody and at ~360 kDa (band 1) and ~160 kDa (band 3) as those for anti-Myc antibody. These results suggest that ALK2 intrinsically associate with ALK4 or ActRIIA or both; especially the band at ~360 kDa (band 1), which was the band with common size detected in Western blot using antibody against Flag, HA or Myc, may indicate receptor complex of ALK2-ALK4-ActRIIA. We also examined effect of activin B on receptor complex; treatment with activin B generally tended to decrease the intensity of slowly migrated-bands (Supplementary Fig. S6B). The reason is currently unknown. It is possible that activin B stimulates internalization of receptor complex; activin A-induced receptor internalization has been reported^39^,^40^. Future studies should clarify the mechanism underlying the decreased band intensity.

### Resistance to LDN-193189 by the formation of an ALK2 and ActRIIA receptor complex

**Figure 4:**
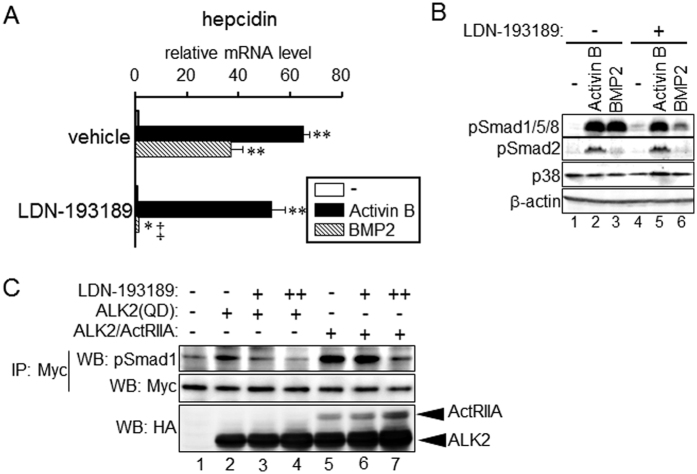
Activin B-mediated signaling is LDN-193189-insensitive. (**A,B**) Rat primary hepatocytes were treated with or without LDN-193189 (100 nM) for 15 min, followed by treatment with activin B (2 nM) or BMP2 (4 nM) for 1 h (**B**) or 8 h (**A**). Expression of hepcidin (**A**) was examined by RT-qPCR analysis. Mean ± SE (n = 3). * and **P < 0.05 and *P* < 0.01, respectively, vs. cells treated with the respective inhibitor but not with activin B. ^‡^*P* < 0.01 vs. cells treated with the respective TGF-β family ligand (none, activin B or BMP2) but not with LDN-193189. (**B**) Phosphorylation of Smad1/5/8 and Smad2 as well as p38 and β-actin as the loading controls was examined by Western blot analysis. (**C**) HepG2 cells were transfected with 6Myc-tagged Smad1 and the indicated HA-tagged ALK2 or ActRIIA. At 4 h post-transfection, cells were treated with or without LDN-193189 (−: 0 nM, +: 100 nM and ++: 400 nM) for 48 h. Smad1 protein expressed in cells was immunoprecipitated by use of an anti-c-Myc antibody, and BR-Smad phosphorylation was examined by Western blot analysis. The cropped images of Western blot analysis are shown because of space liminations; images of the full-length blot are Supplementary Fig. S12.

LDN-193189 is an inhibitor for type I receptors of the BMP pathway, i.e., ALK2, ALK3 and ALK6^15^. Provided that ALK2 is the principle receptor that confers activin B-induced hepcidin transcription, we expected that the up-regulation of hepcidin expression by activin B would be blocked by LDN-193189. BMP2-induced hepcidin expression was blocked by LDN-193189 in primary hepatocytes, but the up-regulation of hepcidin expression by activin B was LDN-193189-resistant (Fig. 4A). Consistent with these results, LDN-193189 did not inhibit activin B-induced Smad1/5/8 phosphorylation, whereas BMP-2-induced phosphorylation of Smad1/5/8 was inhibited by LDN-193189 (Fig. 4B). LDN-193189 decreased basal transcription of hepcidin, but the responsiveness to activin B, i.e., fold-induction of hepcidin transcription due to activin B treatment was not decreased in HepG2 cells. The fold-induction of activin B in cells treated with vehicle and in those treated with LDN-193189 was 5.6 and 16.9, respectively (Supplementary Fig. S7A). By contrast, LDN-193189 inhibited the responsiveness to BMP-2 (5.7-fold and 1.4-fold, respectively, Supplementary Fig. S7A). Similarly, LDN-193189 decreased basal Smad1/5/8 phosphorylation levels. However, LDN-193189-treated cells still responded to activin B but not BMP-2 to induce phosphorylation of Smad1/5/8 (Supplementary Fig. S7B). LDN-193189 did not affect activin B-induced Smad2 phosphorylation in primary hepatocytes (Fig. 4B) and HepG2 cells (Supplementary Fig. S7B).

Failure to inhibit activin B-mediated hepcidin gene induction and phosphorylation of BR-Smad by LDN-193189 clearly contradicts the blockade of activin B-mediated hepcidin transcription by ALK2 knockdown. We hypothesized that ALK2 and ActRIIA complex formation may be responsible for resistance to inhibitory effects of LDN-193189. Overexpression of the type I receptor and the type II receptor induces ligand-independent phosphorylation of R-Smad^41^. Phosphorylation of Smad1 resulting from co-expression of ALK2 and ActRIIA was not inhibited by 100 nM of LDN-193189 in HepG2 cells, although 400 nM of LDN-193189 decreased Smad1 phosphorylation levels in ALK2 and ActRIIA-expressing cells (Fig. 4C, *lane 5–7*). By contrast, Smad1 phosphorylation induced by the expression of ALK2(QD), a constitutively active ALK2^42^, was decreased by 100 nM LDN-193189 (Fig. 4C, *lane 2* and *3*). Consistent with these results, co-expression of ActRIIA blocked the inhibition of ALK2(QD)-mediated hepdicin transcription by LDN-193189 (Supplementary Fig. S7C). Activin B-induced formation of ALK2 and ActRIIA may decrease the inhibitory effect of LDN-193189 on BR-Smad-mediated signaling.

### Activin B-induced activation of the BMP pathway is not limited to liver-derived cells

**Figure 5:**
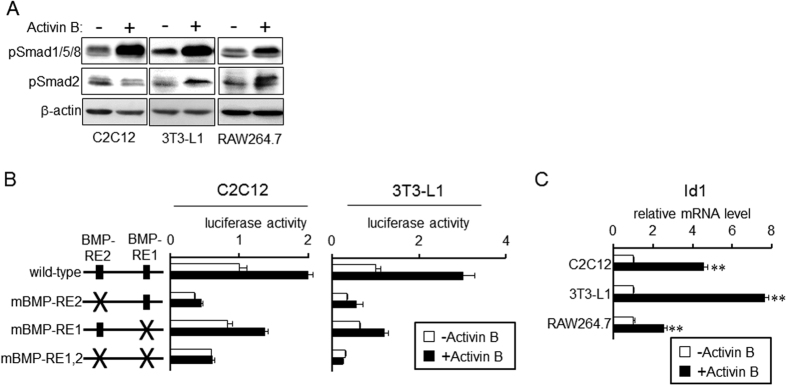
Activin B-induced activation of the BMP pathway is not limited to liver-derived cells. (**A**) C2C12 myogenic cells, 3T3-L1 preadipocytes and RAW264.7 macrophage cells were treated treatment with or without activin B (2 nM) for 1 h. Phosphorylation of Smad1/5/8 and Smad2 as well as β-actin as the loading control was examined by Western blot analysis. (**B**) C2C12 cells or 3T3-L1 cells were transfected with the indicated reporter and CMV-βGal. At 24 h post-transfection, cells were treated with or without activin B (2 nM) for 24 h. Luciferase activity normalized to β-galactosidase activity was calculated, and the relative luciferase activity in cells treated without activin B was set at 1. Mean ± SE (n = 3). (**C**) C2C12, 3T3-L1 and RAW264.7 cells were treated with or without activin B (2 nM) for 4 h (C2C12 and 3T3-L1) or 8 h (RAW264.7). Expression of Id1 was examined by RT-qPCR analysis. The expression level in the control cells treated without activin B was set at 1. Mean ± SE (n = 3 for C2C12 and 3T3-L1, n = 4 for RAW264.7). **P < 0.01, vs. respective cells treated without activin B. The cropped images of Western blot analysis are shown because of space liminations; images of the full-length blot are Supplementary Fig. S13.

We examined whether activin B-induced activation of the BMP pathway is limited to liver-derived cells. Phosphorylation of BR-Smad and AR-Smad in response to activin B was evaluated in C2C12 myogenic cells, 3T3-L1 adipogenic cells and RAW264.7 macrophage cells. As shown in Fig. 5A, activin B induced Smad1/5/8 phosphorylation in all three cell types. Activin B also stimulated phosphorylation of Smad2 in 3T3-L1 cells and RAW264.7 cells but not C2C12 cells. Our previous study also showed inability of activin A to phosphorylate Smad2 in C2C12 cells^43^; it is possible that C2C12 cells are not target cells of activins. Transcription of hepcidin(-2018)-luc was increased by activin B in C2C12 cells and 3T3-L1 cells, which was blocked by BMP-RE mutations (Fig. 5B). Furthermore, activin B also up-regulated the expression of Id1, a BMP signaling target gene^44^, and stimulated the transcription of BRE-luc, a well-known BMP-responsive reporter^45^ (Fig. 5C and Supplementary Fig. S8). These results suggest that activin B-mediated activation of the BMP pathway is not limited to liver-derived cells but applicable to a wide range of cells.

## Discussion

Previously, activin B was thought to be induced in response to hepatic inflammation, and the induced activin B stimulated the expression of the iron-regulating hormone hepcidin^19^,^21^. However, the source of activin B and the detailed induction of hepcidin were unclear. Here, we clarify the signal transduction from sinusoids to parenchyma that is mediated by activin B in response to liver inflammation. Endothelial cells and Kupffer cells produce activin B during LPS-induced inflammation, and the induced activin B stimulates hepcidin production in liver parenchymal cells. ALK2 in concert with ActRIIA transmits the activin B signal to stimulate hepcidin transcription through phosphorylation and activation of BR-Smad. Activin B also stimulates AR-Smad-mediated signaling through ALK4 and ActRIIs, leading to up-regulation of CTGF expression (Supplementary Fig. S9).

Previous studies have reported that hepcidin transcription is regulated by activin B, but conflicting mechanisms were proposed^19^,^21^. Besson-Fournier *et al*.^19^ suggested ALK3 is the primary receptor that induces hepcidin transcription through activin B, whereas ALK2 was proposed to be the main receptor to confer activin B-induced hepcidin transcription^21^. The present results on the transcriptional regulation of hepcidin by activin B are consistent with the results of Canali *et al*.^21^. It is unclear why the previous results are inconsistent. Besson-Fournier *et al*.^19^ concluded that ALK3 transmits activin B-induced hepcidin expression because treatment with the extracellular region of ALK3 fused to an immunoglobulin Fc domain (ALK3-Fc) but not ALK2-Fc decreased the hepcidin expression level in hepatocytes treated with activin B. The interpretation was that activin B was trapped by ALK3-Fc and could not be bound to ALK3 expressed at the cell surface. However, they did not examine hepcidin expression in ALK3-Fc-treated cells without activin B; it is possible that endogenously produced molecule(s) that induces hepcidin expression is trapped by ALK3-Fc. In fact, the present study revealed that ALK3 gene knockdown decreased the basal expression of hepcidin but did not affect responsiveness to activin B (Fig. 3A). ALK3-Fc is expected to also regulate hepcidin expression negatively, and to lead to the unaltered responsiveness to activin B.

There are points that are not consistent with the results of Canali *et al*.^21^. Consistent with previous results^21^,^33^,^34^, activin B induced AR-Smad phosphorylation and AR-Smad-mediated cell responses. Our results suggested that ALK4 confers activin B signaling in liver-derived cells. By contrast, provided that knockdown of ALK7 blunted activin B-induced phosphorylation of AR-Smad, Canali *et al*.^21^ suggested ALK7 as a receptor for activin B in HepB3 cells. However, ALK7 is expressed in a tissue-restricted manner^46^,^47^, and its expression in liver is relatively low^46^,^47^. In fact, ALK7 expression in the liver and primary hepatocytes was below the detection limit in RT-PCR analysis, and it could not be detected in HepG2 cells and Hepa1-6 cells (Fig. 3E). We conclude that AR-Smad phosphorylation and activation mediated by ALK4 in response to activin B is physiologically relevant in liver-derived cells. Canali *et al*.^21^ also suggested that activin B-induced BR-Smad signaling is limited to liver-derived cells. By contrast, our results clearly reveal the activation of the BMP pathway by activin B in several cell types.

In the canonical signal transduction of the TGF-β family described above, members of the TGF-β and activin subgroups signal through inducing AR-Smad phosphorylation, whereas the BMP subgroup signals though BR-Smad phosphorylation. The type I receptor determines the specificity of the signaling; ALK4/5/7 primarily phosphorylate AR-Smad, whereas ALK1/2/3/6 phosphorylate BR-Smad^11^,^12^,^13^. Previous studies have shown three types of non-canonical Smad signaling: (1) transient BR-Smad phosphorylation mediated by activated ALK5 in response to TGF-β^41^,^48^,^49^,^50^, (2) TGF-β-induced transient phosphorylation of BR-Smad by ALK1 in an endothelial cell-restricted manner^51^, and (3) long-lasting AR-Smad phosphorylation by ALK3/6 in response to BMP^41^. In summary, a ligand uses a canonical type I receptor that phosphorylates a non-canonical R-Smad (case 1 and 3). In addition, a ligand can use a different class of receptor that phosphorylates the canonical R-Smad for the type I receptor. As a whole, the non-canonical R-Smads for the ligand are phosphorylated (case 2). The present study reveals that activin B induces prolonged phosphorylation of the non-canonical BR-Smads via ALK2 and stimulates BMP-regulated gene transcription, including hepcidin, not only in hepatocytes but also in non-hepatocytes. These results are consistent with case 2, and activin B induces non-canonical R-Smads mediated by a non-canonical type I receptor. However, BR-Smad phosphorylation was not transient but long-lasting. Therefore, the non-canonical Smad phosphorylation shown here is a unique example.

In the current signaling model of the TGF-β family, the BMP type II receptors partly overlap with those of activin. ActRIIA and ActRIIB act as type II receptors for activin and BMP, although the type I receptors that confer the signal are distinct between activin (ALK4/7) and BMP (ALK2/3/6)^11^,^12^,^13^. The present study indicates intrinsic complex formation of ALK2 with ALK4, and also suggests the inclusion of ActRIIA in the receptor complex. In view of the receptor complex tetramer formation, consisting of two type II receptors and two type I receptors, after ligand binding^11^,^12^,^13^, activin B may bind ALK2 and ALK4 together with ActRIIA and ActRIIB in a receptor complex to signal (Supplementary Fig. S9).

Treatment with the BMP receptor inhibitor LDN-193189 decreased the basal expression of hepcidin in HepG2 cells. However, responsiveness to activin B but not BMP2, i.e., fold-induction of hepcidin transcription in response to activin B, was not decreased in primary hepatocytes and HepG2 cells, respectively (Supplementary Fig. S7A). Responsiveness to the molecules of interest should be evaluated as the fold-induction of expression and transcription in non-treated cells^52^. Thus, we suggest that LDN-193189 simply decreased basal expression and transcription of hepcidin and that LDN-193189-treated cells were still responsive to activin B. Besson-Fournier *et al*.^19^ suggested inhibition of activin B-induced hepcidin expression by LDN-193189 on the basis of the data that hepcidin expression was lower in cells treated with both activin B and LDN-193189 than in those treated with activin B alone. However, similar to the present study, basal expression of hepcidin, i.e., expression in cells treated without activin B, was also decreased by LDN-193189, and responsiveness to activin B was unaffected by LDN-193189. Thus, although the present results on the role of LDN-193189 in hepcidin expression are consistent with the results by Besson-Fournier *et al*.^19^, the interpretation of the results is distinct.

We also showed that the effect of LDN-193189 to inhibit ALK2-mediated phosphorylation of BR-Smad and hepcidin transcription is diminished by ActRIIA expression. Previous studies have shown that in addition to ALK2/3/6 kinase activity^15^, LDN-193189 inhibits activity of ALK4/5^53^ and ActRIIA^54^ serine/threonine kinases as well as the other kinases unrelated to TGF-β family signaling^55^. The present results on resistance of LDN-193189 activity by co-expression of ALK2 and ActRIIA, however, can not be simply explained by inhibition of unintentional kinase activity. It may be difficult for LDN-193189 to approach ALK2 to inhibit the serine/threonine kinase activity of ALK2 after complex formation with ActRIIA. The intracellular domain of ALK2 has higher affinity to ActRIIA than to ActRIIB^56^. It is possible that steady ALK2 and ActRIIA complex formation leads to relative resistance to LDN-193189.

The present study indicates that activin B simultaneously stimulates signaling via AR-Smad and BR-Smad, increasing expression of CTGF and hepcidin, respectively, in hepatocytes. As hepatitis triggers the onset of liver fibrosis^57^, inflammation-induced activin B could be one molecule that links inflammation to fibrosis, which is mediated by the induction of CTGF. In addition, activin B may be responsible for the onset of inflammation-induced anemia through the up-regulation of hepcidin expression. Thus, to prevent adverse effects on liver function, activin B induced by inflammation may be a possible therapeutic or preventive target.

## Materials and Methods

### Materials

Activin A and activin AB^58^ and anti-inhibin α, βA (GY) and βB (G544) antibodies^59^,^60^ were generously provided by Dr. Y. Hasegawa (Kitasato University). Activin A and activin AB were purified near homogeneity from bovine follicular fluid^58^. Amino acid sequence of mature region of bovine inhibin βA and βB is the same as human inhibin βA and βB, respectively. Recombinant human activin B and BMP2 were purchased from R & D Systems (Minneapolis, MN, USA); according to the manufacturer’s instruction, activin B and BMP2 were >90% and >95% purity assessed by SDS-PAGE, respectively. The following reagents were also purchased and used: lipopolysaccharide (LPS) from *Pseudomonas aeruginosa 10* (L8643) was from Sigma (St. Louis, MO, USA); LDN-193189 was from Stemgent (San Diego, CA, USA); A83–01 was from Calbiochem (Darmstadt, Germany); rabbit polyclonal antibodies against phospho-Smad1 (Ser463/Ser465)/Smad5 (Ser463/Ser465)/Smad8 (Ser426/Ser428) (#9511), phospho-Smad2 (Ser465/Ser467) (#3101) and p38 (#9212), and rabbit monoclonal antibody against phospho-Smad3 (Ser423/Ser425) (C25A9, #9520) were from Cell Signaling Technology (Danvers, MA, USA); rat monoclonal antibody against F4/80 (CI:A3-1, ab6640), rabbit polyclonal antibody against CD31 (ab28364), and mouse monoclonal antibody against β-actin (AC-15, ab6276) was from Abcam (Cambridge, MA, USA); a rabbit polyclonal antibody against c-Myc (A-14, sc-789) was from Santa Cruz Biotechnology (Santa Cruz, CA, USA).

### Plasmids

Constitutively active ALK2 and ALK3 were provided by Dr. K. Miyazono. Expression vectors for receptors of the TGF-β family and Smad were previously described^41^. The hepcidin reporter construct, i.e., the hepcidin promoter spanning nt −2018 to nt −35 inserted into the luciferase reporter vector pGL4 (hepcidin(-2018)-luc), and the mutated plasmids were previously described^52^,^61^; the translational initiation site is defined as +1. Cytomegalovirus promoter-controlled β-galactosidase expression vector (CMV-βGal) was used as a plasmid to correct transfection efficiency.

### Animals and cell culture

Animal care and experiments were approved by Animal Care Committee of Kyoto University (27-43-2 and 27–72) and Institutional Animal Care and Use of Committee, Kitasato University (15-036), and all animal experiments were conducted in accordance with the approved guidelines. Primary hepatocytes from male Sprague-Dawley rats were prepared as described previously^61^. HepG2 human hepatoma cells, Hepa1-6 mouse hepatoma cells, C2C12 mouse myoblasts, 3T3-L1 mouse preadipocytes and RAW264.7 mouse macrophage-like cells were cultured in DMEM with 10% heat-inactivated FBS and antibiotics.

### Immunohistochemistry

To identify the localization of inhibin subunits in the liver, male C57BL/6 mice aged 9 wks were intraperitoneally injected with LPS (5 mg/kg) or phosphate-buffered saline (PBS) (n = 4). After 6 h of injection, livers were fixed with Bouin’s solution, embedded in paraffin, and sectioned to 4 μm thickness. Immunohistochemical analyses using anti-inhibin α, βA or βB antibody were performed as described by Asano *et al*.^62^.

### siRNA transfection

HepG2 cells (7.2 × 10^5^ per 60 mm dish) were transfected with 10 μL of Lipofectamine RNAi Max (Invitrogen) and 200 pmol of siRNA according to the manufacturer’s protocol. After 24 h of siRNA transfection, cells were trypsinized, replated into 24-well plates or 12-well plates for 20 h, and used for subsequent experiments. The siRNA sequences are shown in Supplementary Table S1.

### RNA isolation, RT-PCR and RT-quantitative PCR

Total RNA isolation, cDNA synthesis, PCR, and real-time quantitative PCR (qPCR) were performed as described previously^61^ with a modification; KOD-plus neo (TOYOBO, Osaka, Japan) was used as the DNA polymerase for PCR. The oligonucleotide primers used are presented in Supplementary Table S2. The ∆∆Ct method was used to normalize the levels of target transcripts to GAPDH levels^63^.

### Immunoprecipitation and Western blotting

HepG2 cells were transiently transfected with 6Myc-tagged Smad1 with or without expression vectors for the type I receptors and type II receptors of the TGF-β family. At 4 h post-transfection, cells were exposed to LDN-193189 for 48 h. Immunoprecipitation and Western blotting were performed as described previously^64^. The immunoreactive proteins were visualized using an ECL Select Western blotting detection system (GE Healthcare, Buckinghamshire, UK) according to the manufacturer’s protocol.

### Luciferase-based reporter assay

HepG2 cells (6 × 10^4^ per well) or Hepa1-6 cells (1 × 10^5^ per well) seeded onto 24-well plates were transfected with hepcidin(-2018)-luc or the indicated reporter plasmid (0.5 μg), indicated expression plasmid, or empty plasmid (0.5 μg), and β-galactosidase expression plasmid under the control of a cytomegalovirus-derived promoter (pCMV-βGal, 0.1 μg) using polyethylenimine Max reagent (Polysciences, Warrington, PA, USA). After 4 h of transfection, cells were treated with activin B or BMP2 for 12–16 h. Firefly luciferase activity was normalized to β-galactosidase activity as previously described^41^. Relative luciferase activity was calculated as the ratio of luciferase activity to β-galactosidase activity, and the activity in unstimulated cells without treatment with inhibitors was set at 1.

### Statistical analyses

Data are expressed as the mean ± standard error (SE). Gene expression data were log-transformed to provide an approximation of a normal distribution before analyses. Differences between gene expression levels in tissues or cells were examined using unpaired *t*-tests. Differences of *P* < 0.05 were considered significant.

## Additional Information

**How to cite this article**: Kanamori, Y. *et al*. Regulation of hepcidin expression by inflammation-induced activin B. *Sci. Rep.* **6**, 38702; doi: 10.1038/srep38702 (2016).

**Publisher's note:** Springer Nature remains neutral with regard to jurisdictional claims in published maps and institutional affiliations.

## Supplementary Material

Supplementary Information

## Figures and Tables

**Table 1:** Immunolocalization of inhibin subunits.

	Inhibin α		Inhibin βA		Inhibin βB	
	Control	LPS	Control	LPS	Control	LPS
Hepatocytes	+	+	±	±	−	±
Kupffer cells	−	−	−	±	−	+
Hepatic stellate cells	±	±	−	−	−	−
Endothelial cells						
Central vein	−	−	−	−	±	++
Interlobular arteriovenous	−	−	−	−	±	++
Sinusoid	−	−	−	−	−	+
Vessel lumen	±	−	±	±	+	++

−: Negative, ±: Faint staining, +: Moderate staining, ++: Intense staining.
