# Supplementary material for: Regulation of hepcidin expression by inflammation-induced activin B
Source: Sci Rep. 2016 Dec 6;6:38702. doi: 10.1038/srep38702 (PMC5138601; doi:10.1038/srep38702)
Supplement: Supplementary Information [file srep38702-s1.pdf]

Regulation of hepcidin expression by inflammation-induced activin B

Yohei Kanamori, Makoto Sugiyama, Osamu Hashimoto, Masaru Murakami,
Tohru Matsui and Masayuki Funaba

Supplemental methods

Liver cell separation

Liver cell separation was performed by the method of Smedsrød and Pertoft (1985)[1]. A male Sprague-Dawley rat aged 5 wks was intraperitoneally injected with LPS (5 mg/kg). At 2.5 h post-injection, the liver was perfused with HBSS and collagenase to isolate parenchymal cells. The undigested tissue was removed by passing it through a 250 μm mesh filter. After centrifugation at 50 × *g* for 2 min, the supernatant containing non-parenchymal cells was centrifuged at 1350 × *g* for 10 min at 4°C. To isolate Kupffer cells and endothelial cells, cell pellets were resuspended in preservation buffer (10 mM HEPES, pH7.4, 140 mM NaCl, 7 mM KCl and 1% BSA) and layered onto a 2-step LSM gradient (densities of 1.035 and 1.064, respectively), followed by centrifugation at 1350 × *g* for 30 min at 4°C. The interface between the two density cushions, containing endothelial cells and Kupffer cells, and a cell band in the high density layer, containing mainly endothelial cells, was recovered.

Chemical cross-linking and sequential immunoprecipitation and immunoblot

HepG2 cells were transfected with Flag-tagged ALK2, HA-tagged ALK4 and 6Myc-tagged ActRIIA. At 48 h after transfection, cells were washed twice with PBS and incubated for 15 min at room temperature with or without activin B (2 nM). Subsequently, cells were washed with PBS and treated with BS3 (1 mM, Dojindo,

Kumamoto, Japan), a cell-impermeable crosslinker, for 30 min at room temperature. The reactions were quenched by adding glycine to a final concentration of 50 mM. Cells were further incubated for 15 min at room temperature and washed with PBS. The cells were lysed and immunoprecipipated with mouse monoclonal antibody against Flag (M2, Sigma) or control mouse IgG (Sigma), followed by immunoblotting with anti-Flag antibody, anti-HA antibody (3F10, Roche Applied Science) or anti-Myc antibody (A-14, Santa Cruz Biotechnology).

1. Smedsrød, B. & Pertoft, H. Preparation of pure hepatocytes and reticuloendothelial cells in high yield from a single rat liver by means of Percoll centrifugation and selective adherence. *J Leukoc Biol* **38,** 213-230 (1985).

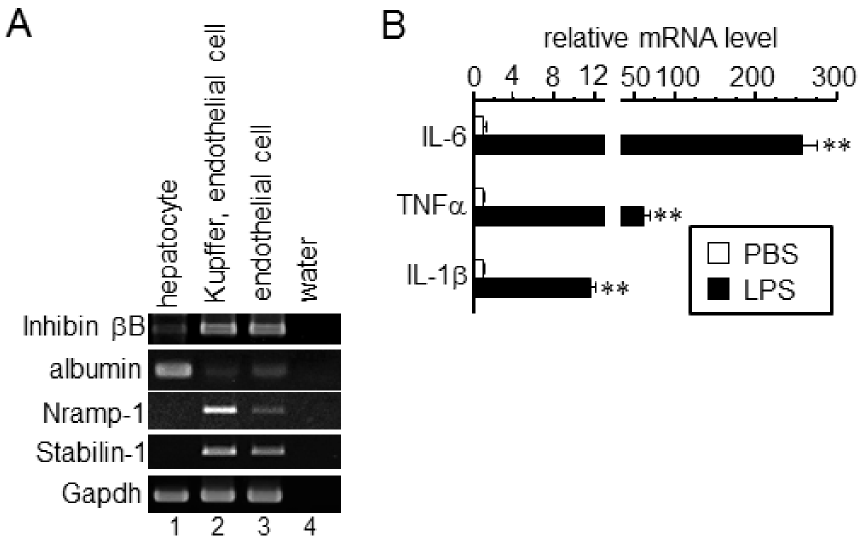

Figure S1. Expression of inhibin βB in isolated cells from LPS-treated livers treated and expression of proinflammatory cytokines in the livers of LPS-treated mice

(A) A male Sprague-Dawley rat aged 5 wks was intraperitoneally injected with LPS (5 mg/kg). At 2.5 h post-injection, liver cells were separated by a centrifugation-based method. Expression of inhibin βB, albumin (hepatocyte marker), Nramp-1 (Kupffer cell marker), stabilin-1 (endothelial cell marker), and Gapdh (housekeeping gene) was examined by RT-PCR. The PCR products were electrophoresed on 2% agarose gels and stained with ethidium bromide. A representative result is shown. (B) C57BL/6 mice were intraperitoneally injected with PBS or with LPS (5 mg/kg). Liver was recovered at 6 h post-injection, and expression levels of proinflammatory cytokines were examined. Gene expression was examined by RT-qPCR analysis, and the expression level in the control mice was set at 1. Mean ± SE (n=4). **: P < 0.01 vs. PBS-treated liver. The cropped images of RT-PCR analysis are shown because of space liminations; images of the full-length gel are Supplementary Fig. S14.

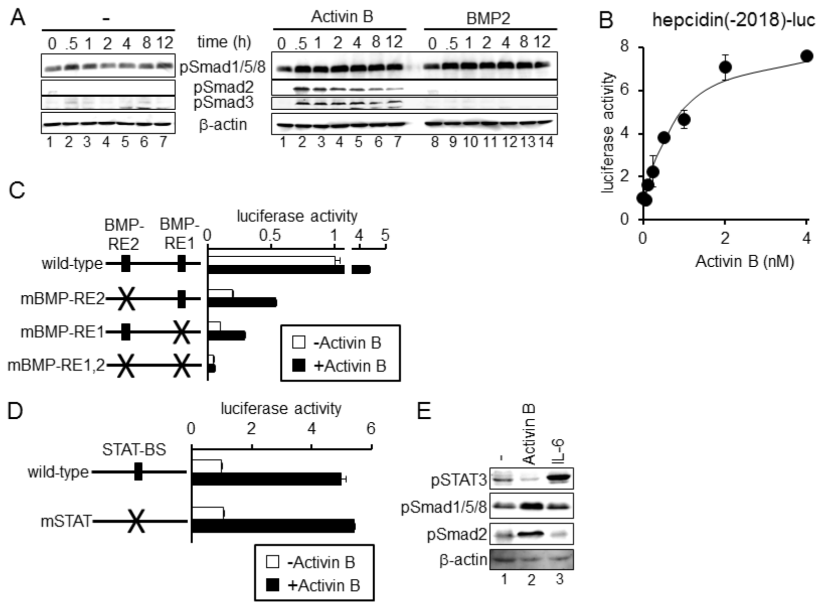

Figure S2. Stimulation of the BMP pathway and hepcidin transcription by activin B via BMP-REs in HepG2 cells

(A) HepG2 cells were cultured in 0.2% FBS for 4 h, followed by treatment with activin B (2 nM) or BMP2 (4 nM) for the indicated time. Phosphorylation of Smad1/5/8, Smad2 and Smad3 as well as the loading control β-actin was examined by Western blot analysis. (B) HepG2 cells were transfected with hepcidin(-2018)-luc and CMV-βGal. At 4 h post-transfection, cells were treated with the indicated concentration of activin B for 16 h. Luciferase activity normalized to β-galactosidase activity was calculated, and the relative luciferase activity in cells transfected with hepcidin(-2018)-luc and treated without activin B was set at 1. Mean ± SE (n=3). (C and D) HepG2 cells were transfected with the indicated reporters and CMV-βGal. At 4 h post-transfection, cells were treated with or without activin B (2 nM) for 12 h. Luciferase activity normalized to β-galactosidase activity was calculated, and the relative luciferase activity in cells transfected with hepcidin(-2018)-luc and treated without activin B was set at 1. Mean ± SE (n=3). (E) HepG2 cells were treated with or without activin B (2 nM) or IL-6 (2.5 nM) for 1 h. Phosphorylation of STAT3, Smad1/5/8 and Smad2 as well as β-actin as the loading control was examined by Western blot analysis. The cropped images of Western blot analysis are shown because of space liminations; images of the full-length blot are Supplementary Fig. S15.

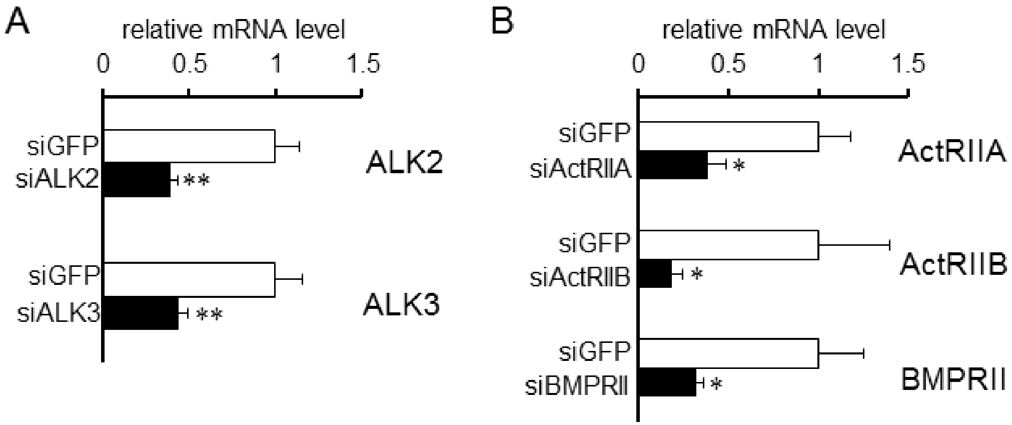

Figure S3. Down-regulation of TGF-β family receptors in HepG2 cells by siRNA transfection

HepG2 cells were transfected with siRNA for the indicated genes. At 48 h of transfection, expression of the type I receptor (A) and type II receptor (B) was examined by RT-qPCR analysis. The expression level in cells transfected with siRNA for GFP was set at 1. Mean ± SE (n=3). * and **: $P < 0.05$ and $P < 0.01$, respectively, vs. cells transfected with siGFP.

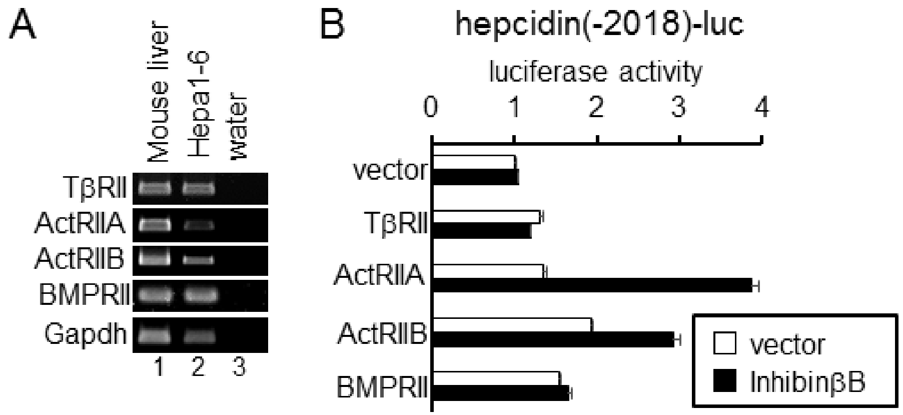

Figure S4. Expression of TGF-β family receptors and recovery of responsiveness to activin B by ActRIIA expression in Hepa1-6 cells

(A) Expression of the TGF-β family receptors in Hepa1-6 cells was examined by RT-PCR. The PCR products were electrophoresed on 2% agarose gels and stained with ethidium bromide. A representative result is shown. (B) Hepa1-6 cells transfected with hepcidin(-2018)-luc and CMV-βGal were co-transfected with or without the indicated expression vector for type II receptor of the TGF-β family. At 4 h post-transfection, cells were treated with or without activin B for 16 h. Luciferase activity normalized to β-galactosidase activity was calculated, and the relative luciferase activity in the control cells treated without activin B was set at 1. Mean ± SE (n=3). The cropped images of RT-PCR analysis are shown because of space liminations; images of the full-length gel are Supplementary Fig. S16.

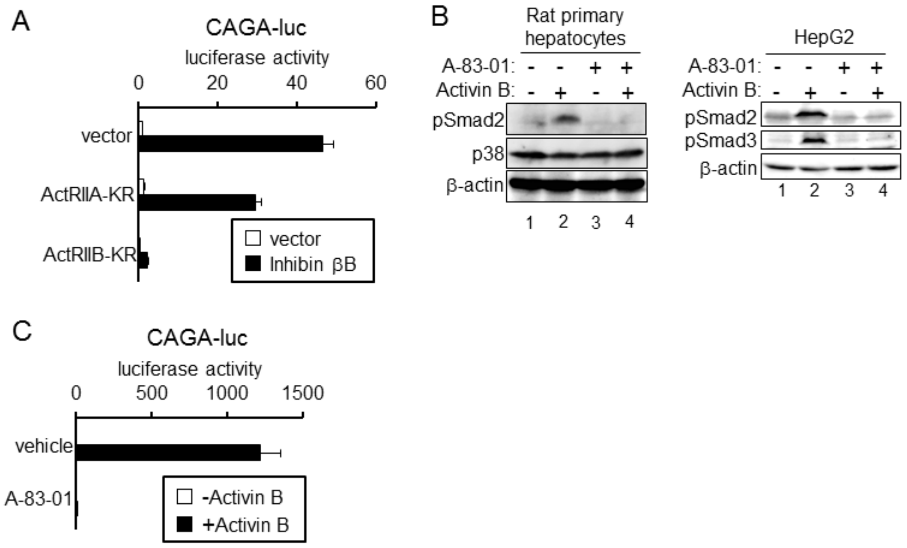

Figure S5. Blockage of activin B-induced AR-Smad-mediated signaling by the expression of dominant-negative ActRIIs and A-83-01 in HepG2 cells

(A) HepG2 cells transfected with CAGA-luc and CMV-βGal were co-transfected with or without expression vectors for inhibin βB (activin B) and the indicated kinase-inactive activin receptor mutant for 16 h. Luciferase activity normalized to β-galactosidase activity was calculated, and the relative luciferase activity in the control cells treated without activin B was set at 1. Mean ± SE (n=3). (B) Rat primary hepatocytes or HepG2 cells were pre-treated with or without A-83-01 (5 μM) for 15 min, followed by treatment with or without activin B (2 nM) for 1 h. Phosphorylation of Smad2 and Smad3 as well as p38 and β-actin as the loading controls was examined by Western blot analysis. (C) HepG2 cells were transfected with CAGA-luc and CMV-βGal. At 4 h of plasmid transfection, cells were treated with or without A-83-01 (5 μM) for 15 min, followed by treatment with activin B (2 nM) for 16 h. Luciferase activity normalized to β-galactosidase activity was calculated, and the relative luciferase activity in the control cells treated without ligand was set at 1. Mean ± SE (n=3). The cropped images of Western blot analysis are shown because of space liminations; images of the full-length blot are Supplementary Fig. S17.

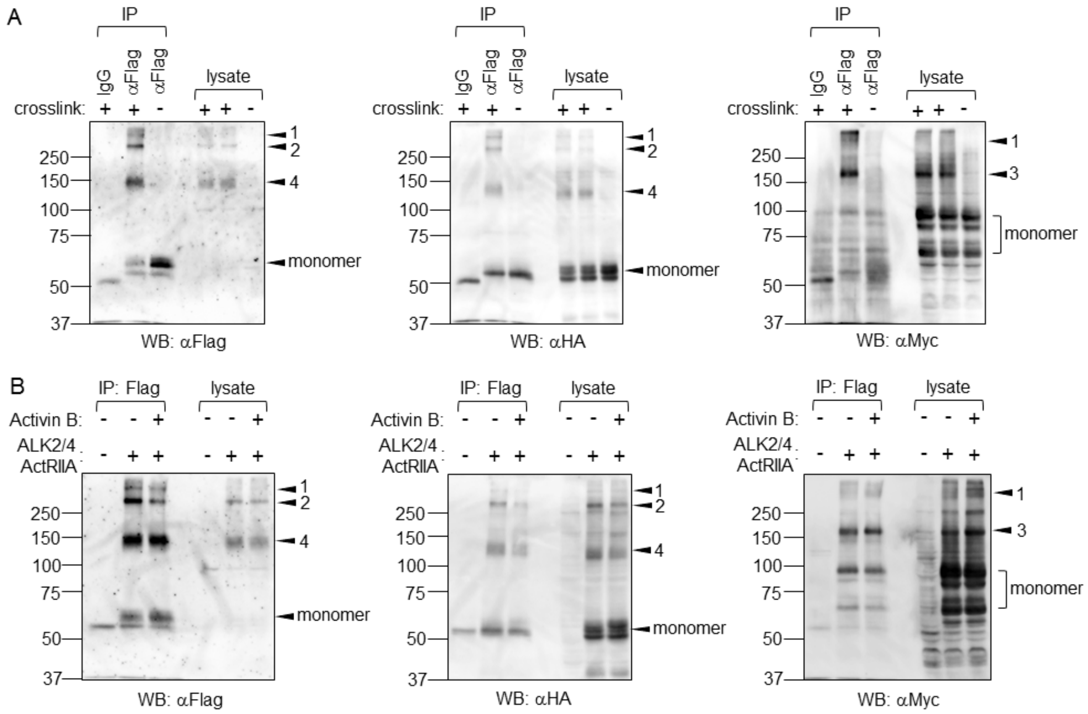

Figure S6. Complex formation of ALK2, ALK4 and ActRIIA in HepG2 cells

(A) HepG2 cells were transfected with Flag-tagged ALK2, HA-tagged ALK4 and 6Myc-tagged ActRIIA, and chemically cross-linked using BS3, a cell-impermeable crosslinker. Cell lysates were immunoprecipitated with anti-Flag antibody or control IgG, followed by Western blot analysis using the indicated antibody. (B) HepG2 cells were transfected with the indicated receptors and treated with or without activin B (2 nM) for 15 min, followed by chemical crosslink with BS3. Cell lysates were sequentially immunoprecipitated with anti-Flag antibody and immunoblot with the indicated antibody. Band 1-4 migrated at ~360 kDa, ~300 kDa, ~160 kDa and ~140 kDa, respectively.

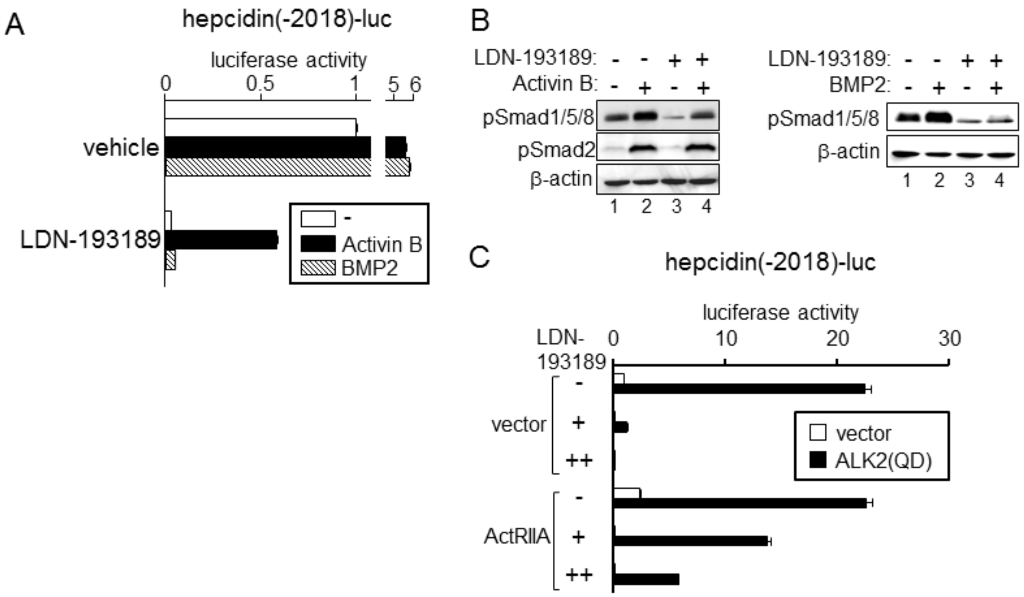

Figure S7. Resistance to LDN-193189 of activin B-induced BR-Smad phosphorylation and ALK2-mediated hepcidin transcription in HepG2 cells

(A) HepG2 cells were transfected with hepcidin(-2018)-luc and CMV-βGal. At 4 h of plasmid transfection, cells were treated with or without LDN-193189 (100 nM) for 15 min, followed by treatment with activin B (2 nM) or BMP2 (4 nM) for 16 h. Luciferase activity normalized to β-galactosidase activity was calculated, and the relative luciferase activity in the control cells treated without ligand was set at 1. Mean ± SE (n=3). (B) HepG2 cells were pre-treated with or without LDN-193189 (100 nM) for 15 min, followed by treatment with or without activin B (2 nM, A) or BMP2 (4 nM, B) for 1 h. Phosphorylation of Smad1/5/8 and Smad2 as well as β-actin as the loading control was examined by Western blot analysis. (C) HepG2 cells were transfected with hepcidin(-2018)-luc and CMV-βGal. At 4 h of post-transfection, cells were treated with or without LDN-193189 (-: 0 nM, +: 100 nM and ++: 400 nM) for 24 h. Luciferase activity normalized to β-galactosidase activity was calculated, and the relative luciferase activity in the control cells treated without ligand was set at 1. Mean ± SE (n=3). The cropped images of Western blot analysis are shown because of space liminations; images of the full-length blot are Supplementary Fig. S18.

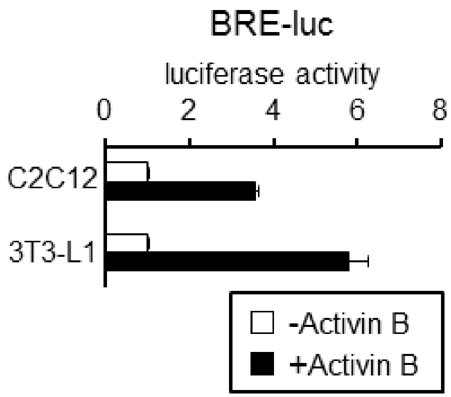

Figure S8. Activin B-mediated transcriptional activation of BRE-luc in C2C12 cells and 3T3-L1 cells

C2C12 cells or 3T3-L1 cells were transfected with BRE-luc and CMV-βGal. At 4 h of plasmid transfection, cells were treated with or without activin B (2 nM) for 16 h. Luciferase activity normalized to β-galactosidase activity was calculated, and the relative luciferase activity in cells treated without activin B was set at 1. Mean ± SE (n=3).

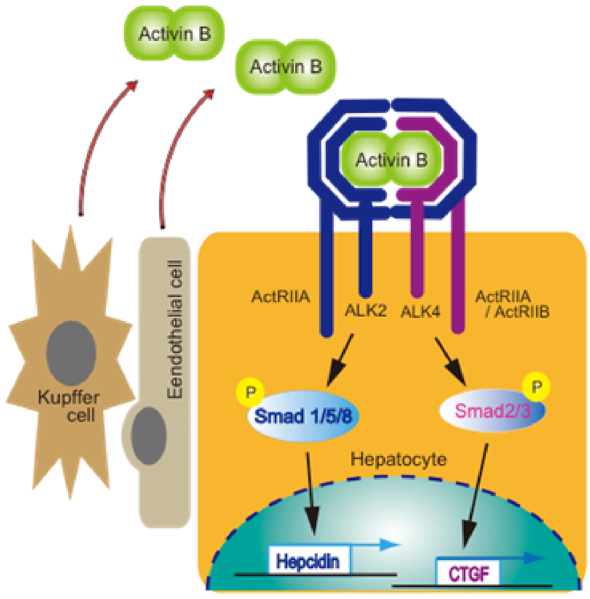

Figure S9. Hypothetical model of activin B function in the liver

Activin B is expressed in vascular endothelial cells and Kupffer cells in response to hepatic inflammation. The induced activin B stimulates phosphorylation of Smad1/5/8 and Smad2/3 though ALK2/ActRIIA and ALK4/ActRIIs, respectively. As a result, activin B induces up-regulation of hepcidin and CTGF expression.

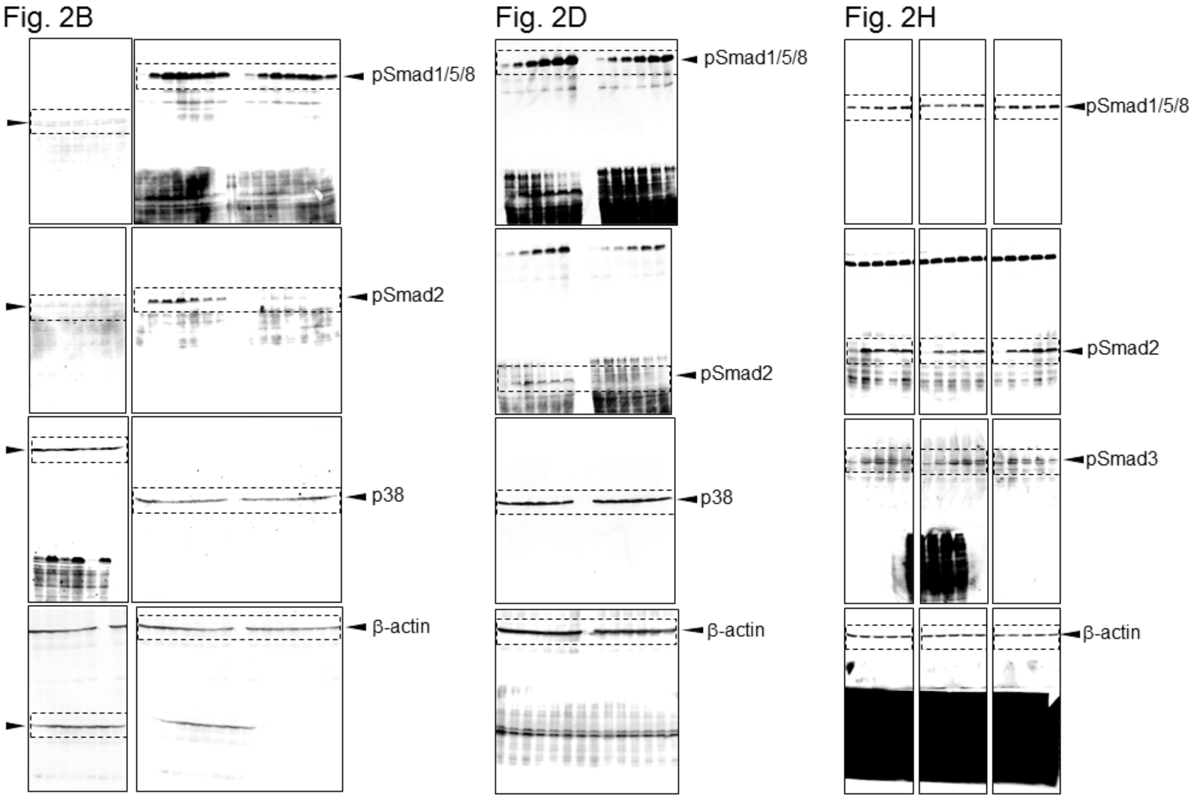

Figure S10. Images of full-length blot shown in Fig. 2

Western blot analysis was visualized by chemical luminescence-based method. The raw results of Western blot analysis are shown; we usually performed Western blot of two membranes, so that the images of full-length are shown also results of unrelated blot. Dashed squares were cropped and shown in Fig. 2.

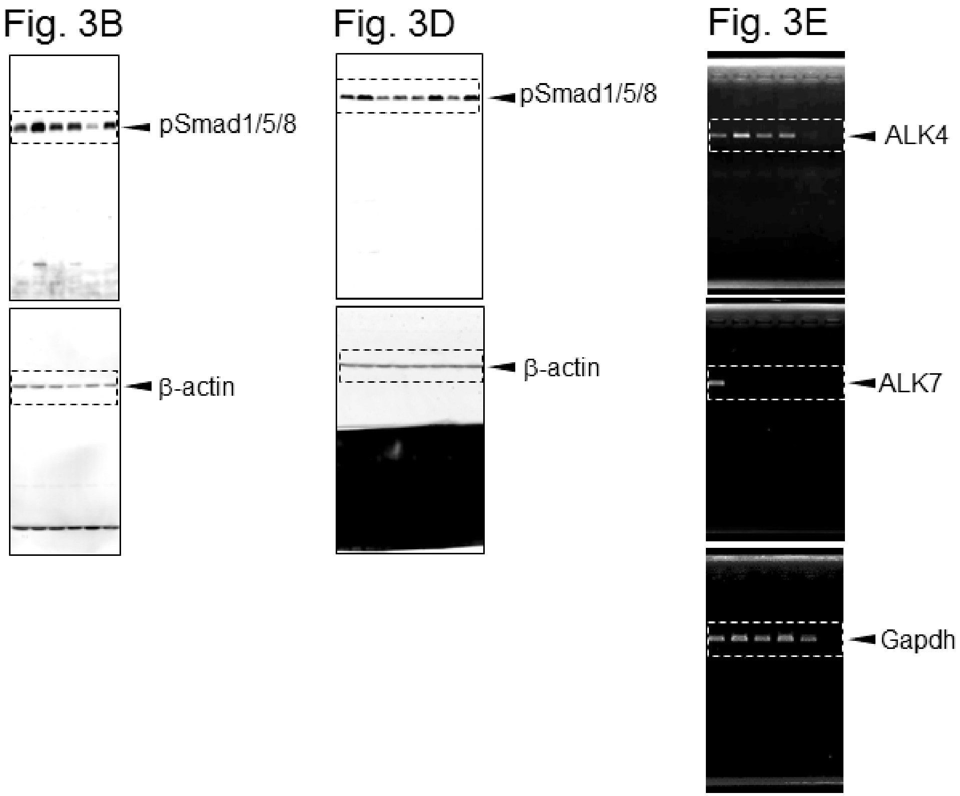

Figure S11. Images of full-length blot and gel shown in Fig. 3

Western blot analysis was visualized by chemical luminescence-based method. The raw results of Western blot analysis are shown; we usually performed Western blot of two membranes, so that the images of full-length are shown also results of unrelated blot. Dashed squares were cropped and shown in Fig. 3.

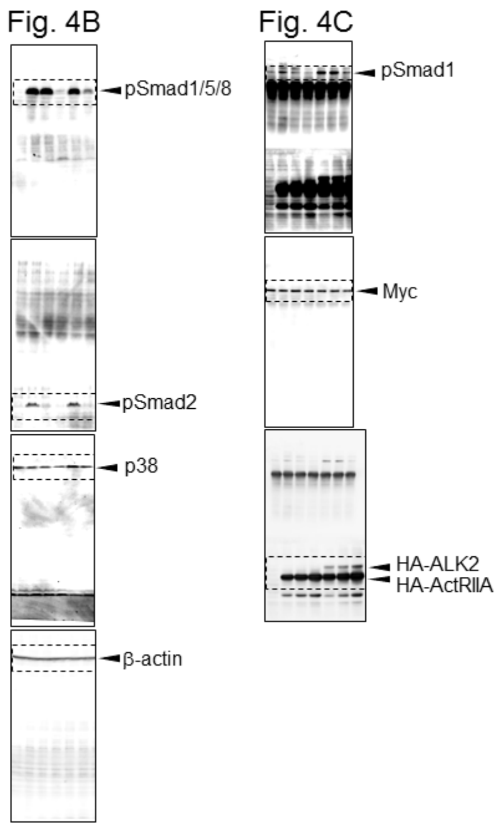

Figure S12. Images of full-length blot shown in Fig. 4

Western blot analysis was visualized by chemical luminescence-based method. The raw results of Western blot analysis are shown; we usually performed Western blot of two membranes, so that the images of full-length are shown also results of unrelated blot. Dashed squares were cropped and shown in Fig. 4.

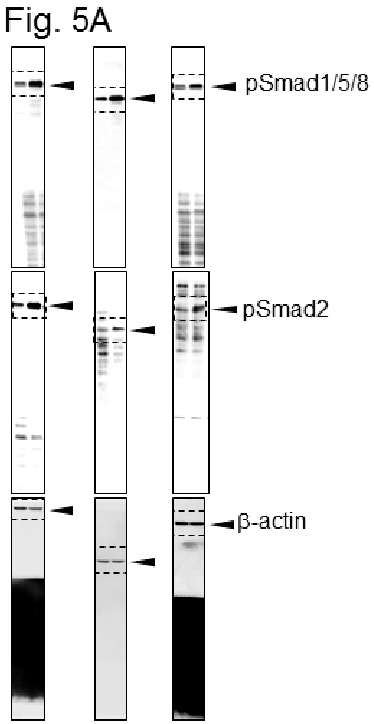

Figure S13. Images of full-length blot shown in Fig. 5

Western blot analysis was visualized by chemical luminescence-based method. The raw results of Western blot analysis are shown; we usually performed Western blot of two membranes, so that the images of full-length are shown also results of unrelated blot. Dashed squares were cropped and shown in Fig. 5.

Fig. S1A

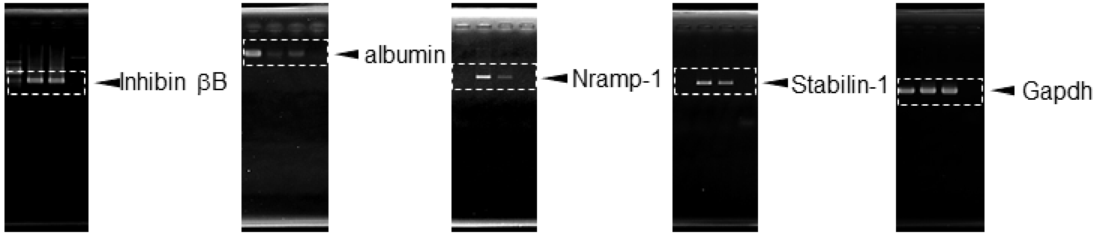

Figure S14. Images of full-length gel shown in Fig. S1

Dashed squares were cropped and shown in Fig. S1.

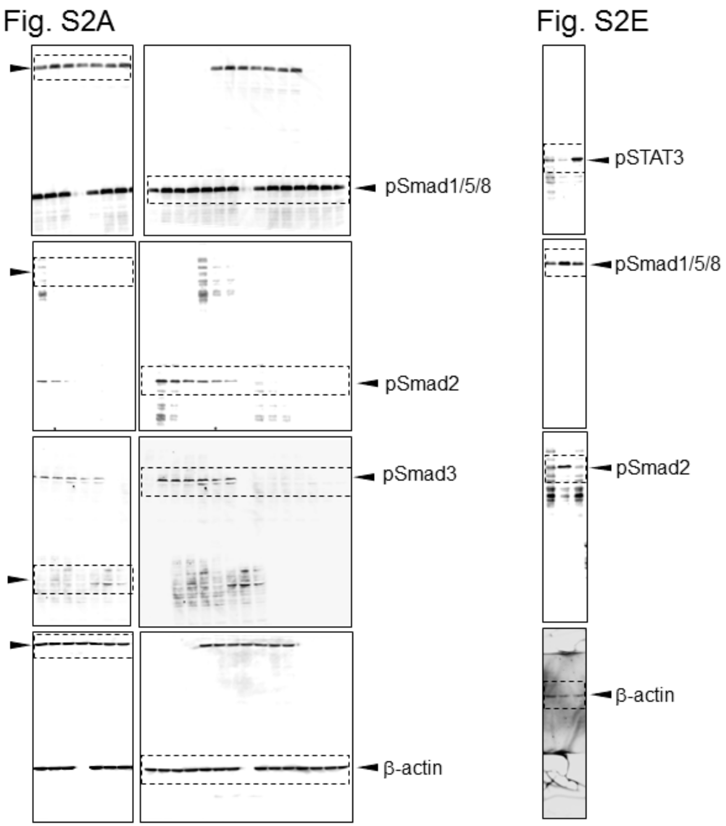

Figure S15. Images of full-length blot shown in Fig. S2

Western blot analysis was visualized by chemical luminescence-based method. The raw results of Western blot analysis are shown; we usually performed Western blot of two membranes, so that the images of full-length are shown also results of unrelated blot. Dashed squares were cropped and shown in Fig. S2.

Fig. S4A

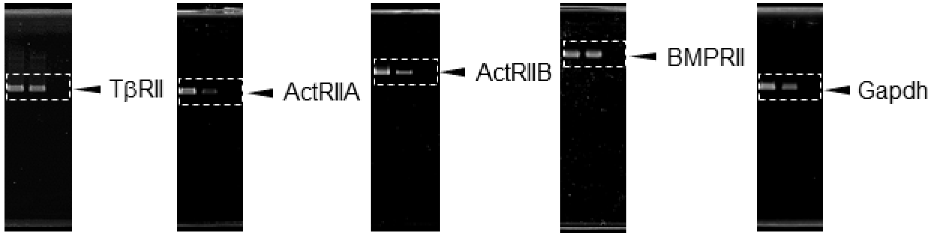

Figure S16. Images of full-length gel shown in Fig. S4

Dashed squares were cropped and shown in Fig. S4.

Fig. S5B

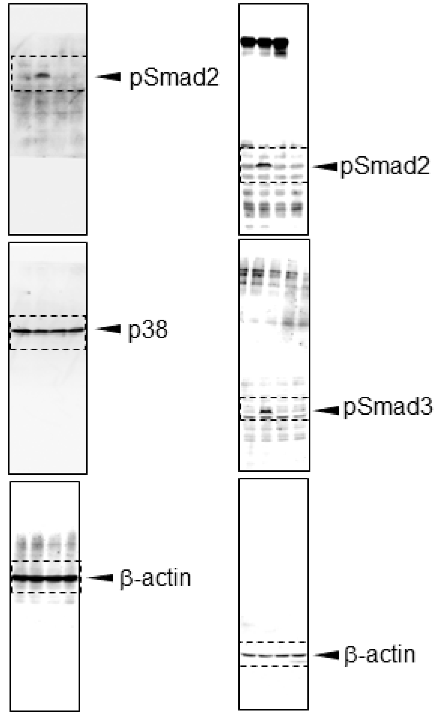

Figure S17. Images of full-length blot shown in Fig. S5

Western blot analysis was visualized by chemical luminescence-based method. The raw results of Western blot analysis are shown; we usually performed Western blot of two membranes, so that the images of full-length are shown also results of unrelated blot. Dashed squares were cropped and shown in Fig. S5.

Fig. S7B

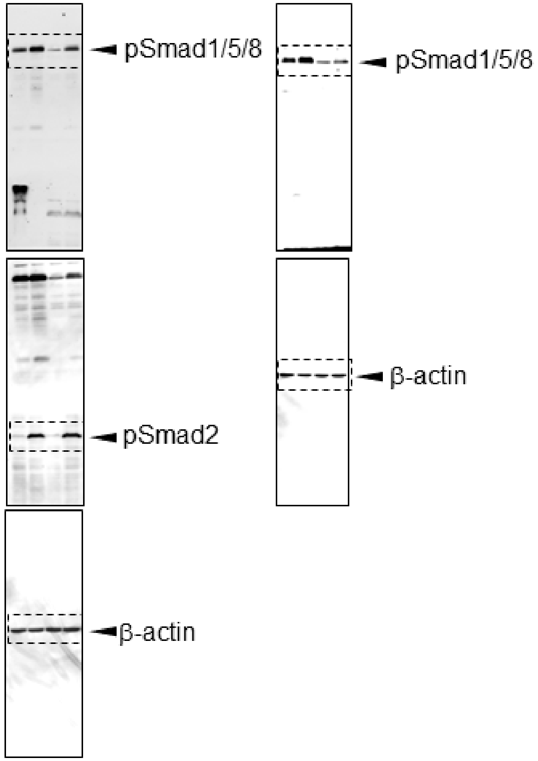

Figure S18. Images of full-length blot shown in Fig. S7

Western blot analysis was visualized by chemical luminescence-based method. The raw results of Western blot analysis are shown; we usually performed Western blot of two membranes, so that the images of full-length are shown also results of unrelated blot. Dashed squares were cropped and shown in Fig. S7.

Supplementary Table S1. Nucleotide sequences used for dsRNAi

Gene	Nucleotide sequence	GenBank accession number
GFP (Control)	5′-GUUCAGCGUGUCCGGCGAG$_d$T$_d$T-3	AB296083
ALK2	5′-GCAGCGAUUACCUGGAUAU$_d$T$_d$T-3	NM_001105
ALK3	5′-AUGGUAUAGUGGAAGAUUA$_d$T$_d$T-3′	NM_004329
ActRIIA	5′-UCAGCGAACUAUUGCCAAA$_d$T$_d$T-3′	NM_001616
ActRIIB	5′-UGAAGUCCUUGACGAGACA$_d$T$_d$T-3′	NM_001106
BMPRII	5′-GAAAGGAAUGAGUGUAAUA$_d$T$_d$T-3′	NM_001204

Supplementary Table S2. Nucleotide sequence of primers used in RT-PCR and RT-qPCR analyses

Gene	Forward primer	Reverse primer	GenBank accession number
RT-PCR:			
hmrALK4[1]	5'- tccttcttccccccttgttgtcc-3'	5'- tgtctttggagagacacatctc -3'	NM_004302 (human)<br>NM_007395 (mouse)<br>NM_199230 (rat)
hmrALK7	5'- gatgtggctgtgaaaatatt -3'	5'- tcttggttcccactttagga -3'	NM_001111031 (human)<br>NM_001033369 (mouse)<br>NM_139090 (rat)
hmrGapdh	5'- ttcattgacctcaactacatggt -3'	5'- gctaagcagttggtggtgcagga -3'	NM_002046 (human)<br>NM_008084 (mouse)<br>NM_017008 (rat)
mActRIIA	5'- tggatgatatcaactgcta -3'	5'- aggagggtaggccatcttgt -3'	NM_007396
mActRIIB	5'- actacaacgccaactgggag -3'	5'- caggttggagcctcgtttct -3'	NM_007397
mBMPRII	5'- tgggaggtgtttatgaggtgt -3'	5'- gaaaagccatctggtaatctgg -3'	NM_007561
mTβRII	5'- cctactctgtctgtggatgacct -3'	5'- atctggatgccctggtggttgag -3'	NM_009371

rAlbumin	5’- tcccagacaaggagaagcag -3’	5’- tcaccgtcttcagctgatctt -3’	NM_134326
rInhibin βB	5’- catggtcacggccctgcgca -3’	5’- ggccagctcctggcagctgt -3’	NM_080771
rNramp-1	5’- atcctcctctggctgaccat -3’	5’- gtgcaaatcgagaccatcgc -3’	NM_001031658
rStabilin-1	5’- ctgcaaagggttctttgggc -3’	5’- cagcttacatgtgcatcggc -3’	XM_008771055

RT-qPCR:

hActRIIA	5’- aagtctgcaggcgataccc -3’	5’- aatgcatccctttggaagttt -3’	NM_001616
hActRIIB	5’- tgtcaagatcttcccactcca -3’	5’- catgccaggtgtgctgaa -3’	NM_001106
hALK2	5’- cgtggagtatggcactatcg -3’	5’- cctgatgtacacgaatgatcca -3’	NM_001105
hALK3	5’- caaagctgtttggagaaaatca -3’	5’- cgcaagcacttgacaatgac -3’	NM_004329
hBMPRII	5’- tctttcagccacaaatgtcct -3’	5’- tgccatcttgtgttgactcac -3’	NM_001204
hTβRII	5’- agcatcacctccatctgtga -3’	5’- tgttctcgtcattctttctcca -3’	NM_001024847
mHprt1	5’- tgcctatgtctcagcctcttc -3’	5’- gaggccatttgggaacttct -3’	NM_013556
mId1	5’- tgctactcacgcctcaagga -3’	5’- tgcaggatctccaccttgct -3’	NM_010495
mIL-1β	5’- tgtaatgaaagacggcacacc -3’	5’- tcttctttgggtattgcttgg -3’	NM_008361
mIL-6	5’- ctctgcaagagacttccatccagt -3’	5’- cgtggttgtcaccagcatca -3’	NM_001314054
mInhibin α	5’- atgcacaggacctctgaacc -3’	5’- tggctggtcctcacaggt -3’	NM_010564

mInhibin βA	5’- aggcacctctgggtcctc -3’	5’- ccctcatgcggtagtggtta -3’	NM_008380
mInhibin βB	5’- cgagatcatcagctttgcag -3’	5’- ggttgccttcattagagacga -3’	NM_008381
mInhibin βC	5’- tcatcagctttgctgacaca -3’	5’- ttctaccagagaagtggaactcg -3’	NM_10565
mInhibin βE	5’- catcagctttgctaccatcataga -3’	5’- aggtggtgggaccaaagag -3’	NM_008382
mTNFα	5’- tgcctatgtctcagcctcttc -3’	5’- gaggccatttgggaacttct -3’	NM_01278601
rCTGF	5’- gctgacctagaggaaaacattaaga -3’	5’- ccggtaggtcttcacactgg -3’	NM_022266
rGapdh	5’- cgtgttcctacccccaatgt -3’	5’- tgtcatcatacttggcaggtttc -3’	NM_017008
rHepcidn	5’- gatggcactcagcactgga -3’	5’- gctgcagctctgtagtctgtct -3’	NM_053469

[1]Small characters shown before the gene name indicate animal species; h: human, m: mouse, r: rat.
